# Early Complications Following Oesophagectomy for Cancer in Relation to Long-Term Healthcare Utilisation: A Prospective Population-Based Cohort Study

**DOI:** 10.1371/journal.pone.0121080

**Published:** 2015-03-13

**Authors:** Eva Doorakkers, Peter Konings, Fredrik Mattsson, Jesper Lagergren, Nele Brusselaers

**Affiliations:** 1 Upper Gastrointestinal Surgery, Department of Molecular medicine and Surgery, Karolinska Institutet, Stockholm, Sweden; 2 Department of Surgery, Radboud University Medical Center, Nijmegen, the Netherlands; 3 Division of Cancer Studies, King’s College London, London, United Kingdom; University Hospital Oldenburg, GERMANY

## Abstract

**Background:**

Little is known about how early postoperative complications after oesophagectomy for cancer influence healthcare utilisation in the long-term. We hypothesised that these complications also increase healthcare utilisation long after the recovery period.

**Methods:**

This was a prospective, nationwide Swedish population-based cohort study of patients who underwent curatively intended oesophagectomy for cancer in 2001-2005 and survived at least 1 year postoperatively (n = 390). Total days of in-hospitalisation, number of hospitalisations and number of visits to the outpatient clinic within 5 years of surgery were analysed using quasi-Poisson models with adjustment for patient, tumour and treatment characteristics and are expressed as incidence rate ratios (IRR) and 95% confidence intervals (CI).

**Results:**

There was an increased in-hospitalisation period 1-5 years after surgery in patients with more than 1 complication (IRR 1.5, 95% CI 1.0-2.4). The IRR for the number of hospitalisations by number of complications was 1.1 (95% CI 0.7-1.6), and 1.2 (95% CI 0.9-1.6) for number of outpatient visits in patients with more than 1 complication. The IRR for in-hospitalisation period 1-5 years following oesophagectomy was 1.8 (95% CI 1.0-3.0) for patients with anastomotic insufficiency and 1.5 (95% CI 0.9-2.5) for patients with cardiovascular or cerebrovascular complications. We found no association with number of hospitalisations (IRR 1.2, 95% CI 0.7-2.0) or number of outpatient visits (IRR 1.3, 95% CI 0.9-1.7) after anastomotic insufficiency, or after cardiovascular or cerebrovascular complications (IRR 1.2, 95% CI 0.7-1.9) and (IRR 1.1, 95% CI 0.8-1.5) respectively.

**Conclusion:**

This study showed an increased total in-hospitalisation period 1-5 years after oesophagectomy for cancer in patients with postoperative complications, particularly following anastomotic insufficiency.

## Introduction

The incidence of oesophageal cancer is increasing in many Western countries.[1] The curatively intended treatment for oesophageal cancer typically includes oesophagectomy, an extraordinarily extensive procedure which holds a 30% to 50% risk of serious postoperative complications, and a 5% risk of in-hospital mortality.[1,2] The overall prognosis for patients with oesophageal cancer is slowly improving, but the relative overall 5-year survival is still only 10–15% and the postoperative 5-year survival 30–55%.[2] Major surgical complications negatively influence survival, reduce quality of life,[3,4] and increase the risk of several symptoms persisting up to 5 years following surgery.[5,6] It is relevant for healthcare administrators and healthcare professionals to understand patterns in healthcare utilisation, which might help predict future costs and healthcare consumption.[7] Studies on cancer survivorship and healthcare utilisation show that compared to the background population, cancer patients more often visit their medical specialist and are more often admitted to the hospital.[7,8,9,10] In-hospitalisation results in the greatest cost allocation for diseases in general.[7] Length of stay at the time of surgery is obviously longer for patients with complications,[11,12] but any influence of early postoperative complications on healthcare utilisation from a longer-term perspective is unknown. Therefore, the main aim of this study was to assess the influence of early major postoperative complications after oesophagectomy for cancer on postoperative healthcare utilisation 1 to 5 years after surgery by using a prospective population-based cohort, taking into account any influence of major patient and tumour characteristics.

## Materials and Methods

### Design

This was a nationwide Swedish prospective and population-based cohort study; the cohort has been described in more detail elsewhere.[13,14] In brief, the entire cohort consisted of 616 patients (90% of all those eligible) who underwent oesophageal resection for cancer of the oesophagus or oesophago-gastric junction with curative intent during the period April 2001 to December 2005 in Sweden.[15] The follow-up period was set at a maximum of 5 years to assess healthcare utilisation related to the oesophageal cancer or its treatment, rather than healthcare use for other reasons (although all healthcare utilisation during the study period was evaluated). This study was organised through collaboration in Sweden between 174 hospital departments involved in the diagnosis or treatment of these patients.[14] Exclusions were made for unknown histology (n = 9) and death within 1 year of surgery (n = 217), since our main interest was long-term healthcare utilisation and healthcare utilisation is expected to be disproportionally increased in patients who die shortly after surgery. Data were collected on patient, tumour and treatment characteristics from the review of medical records from all Swedish hospitals that performed oesophagectomies during the study period. This included age, sex, comorbidity, histological tumour type and tumour stage, as well as the exposure data on pre-defined complications (presented below).[14] Outcome data on hospitalisation dates, days of in-hospitalisation and visits to the outpatient clinic of the study cohort members following the initial hospital stay during surgery were collected from the nationwide complete Swedish Patient Registry.

### Exposures

The study exposure was pre-defined significant complications within 30 days of surgery. The complications were selected by a group of experienced Swedish oesophageal surgeons and researchers prior to the inclusion phase of the cohort.[14,16] The following complications with these definitions were included: 1) major postoperative bleeding (exceeding 2L or requiring reoperation), 2) anastomotic insufficiency (clinically obvious and radiologically or endoscopically verified), 3) necrosis of the substitute (clinically significant ischemia entailing perforation or ulceration), 4) intra-abdominal abscesses (causing clinical symptoms or radiologically verified), 5) intra-thoracic abscesses (causing clinical symptoms or radiologically verified), 6) sepsis (causing clinical symptoms and positive bacterial culture in the blood), 7) wound infection (causing clinical symptoms and requiring intervention), 8) anastomotic leakage (causing clinical symptoms and verified by radiology or endoscopy), 9) kidney failure (requiring renal replacement therapy), 10) respiratory insufficiency (requiring re-intubation and mechanical ventilation), 11) liver insufficiency (causing jaundice), 12) recurrent laryngeal nerve paralysis (ascertained by laryngeal inspection), 13) pneumonia (proven by infiltrate on radiology and causing clinical symptoms with fever, coughing or dyspnoea), 14) lung embolism (radiologically verified and requiring treatment), 15) other embolism (radiologically verified and requiring treatment), 16) deep venous thrombosis (clinically or radiologically verified and requiring treatment), 17) ileus (radiologically verified and requiring surgery), 18) injured ductus thoracicus (with severe lymph leakage requiring drainage for more than 7 days or reoperation), 19) acute myocardial infarct (clinical symptoms and verified with electrocardiogram or heart enzymes), 20) atrial fibrillation (requiring treatment and new-onset atrial fibrillation), 21) cerebrovascular infarct (verified by computerised tomography), 22) strictures of anastomosis (requiring endoscopic intervention), and 23) gastric perforation (requiring surgical intervention).

The main exposure, number of complications, was categorised into three groups: 1) no complications, 2) 1 complication, or 3) more than 1 complication. The secondary exposures were two specific groups of complications, which were expected to be associated with an increased healthcare utilisation in the long-term, namely cardiovascular or cerebrovascular complications (including embolism, deep venous thrombosis, myocardial infarction, atrial fibrillation and cerebrovascular infarction) and anastomotic insufficiency (also including necrosis of the substitute or anastomotic leakage). The group of cardiovascular or cerebrovascular complications was categorised in 3 groups, 1) no complications, 2) complications other than cardiovascular or cerebrovascular complications, or 3) cardiovascular or cerebrovascular complication(s). Anastomotic insufficiency was categorised as 1) no complications, 2) complications other than anastomotic insufficiency or 3) anastomotic insufficiency (as defined above).

### Outcome

Three outcome variables were assessed from the Patient Registry: 1) total number of days of in-hospitalisation, 2) number of hospitalisations, and 3) number of outpatient visits to a specialist clinic. The study period was categorised into two periods: 1) the first year after surgery, excluding the oesophagectomy hospitalisation, and 2) the period from 1 year up to 5 years after oesophagectomy. Indications for hospitalisations or outpatient visits were not taken into account.

### Confounding

Possible confounders that were taken into account in the statistical models were: 1) age (categorised into 3 groups: <60, 60–74, or ≥75 years), 2) sex, 3) comorbidity (3 groups: 0, 1, >1; including hypertension, angina pectoris, heart failure, chronic obstructive pulmonary disease/emphysema, asthma, diabetes, smoking, kidney diseases, liver diseases, other cancers and other diseases), 4) tumour stage (4 groups: 0-I, II, III or IV, according to 6^th^ edition of the TNM classification[17]), 5) histological type of tumour (2 groups: adenocarcinoma or squamous cell carcinoma), 6) annual hospital volume of oesophagectomies (2 groups: <15 or ≥15 oesophagectomies per year),[18] and 7) neoadjuvant therapy (2 groups: yes or no).

### Statistical analysis

Quasi-Poisson models were used to measure associations between the exposure and outcome variables. Such models took over-dispersion into account and adjusted for all above mentioned confounders. These models were used to account for the fact that events in this study did not occur independently as assumed in conventional Poisson models, but were clustered within individual patients and within groups of patients. We used log(number of days at risk) as an offset in the models. The risk estimates were expressed as incidence rate ratios (IRR) and 95% confidence intervals (95% CIs). There was a low number of missing values (n = 2 (0.5%), in one covariate) which was unlikely to influence modelling results; hence we opted for a complete case analysis. The baseline in these models was a patient with the following characteristics: no complications, age younger than 60 years, male sex, no comorbidity, tumour stage 0-I, adenocarcinoma, hospital volume of <15 oesophagectomies per year, and no neoadjuvant therapy. Since patients who died during the study period had a shorter time at risk for the occurrence of events, we used an offset in the models. This is equivalent to using the event rate. The hospitalisation for oesophagectomy was excluded from the analysis for all patients. All statistical analyses were performed using R.[19]

The unadjusted survival rates for each exposure group are visualized by means of a Kaplan Meier curve.

### Ethical considerations

All patients gave informed consent before inclusion in the study. The study was approved by the Regional Ethical Review Board in Stockholm, Sweden.

## Results

### Patients

**Fig 1:**
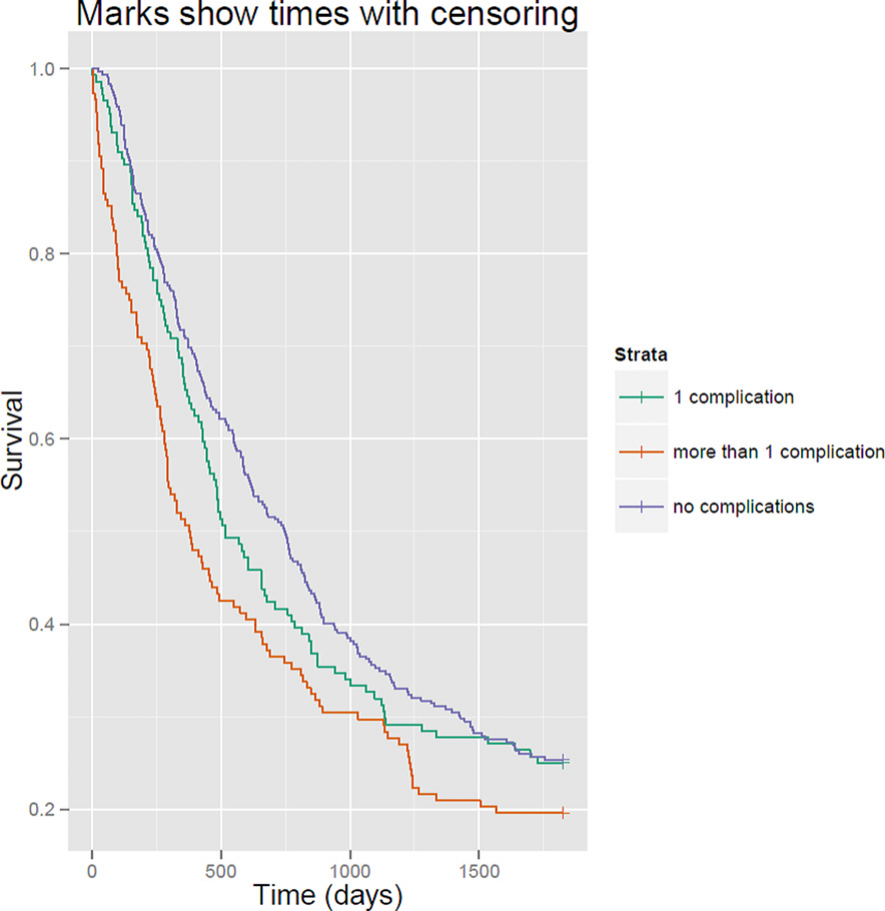
Kaplan Meier curve showing survival time in days by exposure group.

Characteristics of the 390 patients are presented in Table 1. There were 221 (57%) patients without complications, 94 (24%) patients with one complication and 75 (19%) patients with more than one complication. In total, 59 patients (15%) had a cardiovascular or cerebrovascular complication and 36 had an anastomotic insufficiency (9%). Relatively more complications were seen in patients with several comorbidities and with early tumour stage. There were no other major differences in baseline patient characteristics (Table 1). The survival time was longer for patients without complications, compared to patients with one or more than one complication (Table 1, Fig. 1). The unadjusted healthcare utilisation in different patient groups, showing minor differences only, is also presented in Table 1.

**Table 1:** Patient characteristics among 390 patients treated with curatively intended oesophagectomy for cancer who survived for at least 1 year.

	Number of complications*			Total
	*0*	*1*	*>1*	
**Total (N (%))**	221 (57)	94 (24)	75 (19)	390 (100)
**Age (years)**				
*<60*	59 (27)	24 (26)	22 (29)	105 (27)
*60–74*	125 (57)	51 (54)	43 (57)	219 (56)
*≥75*	37 (17)	19 (20)	10 (13)	66 (17)
**Sex**				
*Male*	173 (78)	77 (82)	62 (83)	312 (80)
*Female*	48 (22)	17 (18)	13 (17)	78 (20)
**Comorbidities (number)**				
*0*	77 (35)	34 (36)	18 (24)	129 (33)
*1*	88 (40)	31 (33)	28 (37)	147 (38)
*>1*	56 (25)	29 (31)	29 (39)	114 (29)
**Tumour stage^**				
*0-I*	43 (19)	22 (24)	25 (34)	90 (23)
*II*	81 (37)	34 (37)	18 (24)	133 (34)
*III*	86 (39)	28 (30)	23 (31)	137 (35)
*IV*	11 (5)	9 (10)	8 (11)	28 (7)
**Histological type of tumour**				
*Adenocarcinoma*	170 (77)	75 (80)	52 (69)	297 (76)
*Squamous cell carcinoma*	51 (23)	19 (20)	23 (31)	93 (24)
**Hospital volume**				
*<15 oesophagectomies/year*	145 (66)	60 (64)	52 (69)	257 (66)
*≥15 oesophagectomies/year*	76 (34)	34 (36)	23 (31)	133 (34)
**Neoadjuvant therapy**				
*Yes*	25 (11)	12 (13)	6 (8)	43 (11)
*No*	196 (89)	82 (87)	69 (92)	347 (89)
**Survival time in days**				
*Median*	747	516	378.5	
*Interquartile range*	324–1826	267–1751	150–1231	
**Discharge to 1 year after oesophagectomy**				
*Days of in-hospitalisation (interquartile range)*	5 (0–16)	8 (0–20)	9 (0–25)	
*Number of hospitalisations (interquartile range)*	1 (0–2)	1 (0–2)	1 (0–3)	
*Number of outpatient visits (interquartile range)*	5 (3–9)	6 (4–10)	6 (3–10)	
**Year 1–5 after oesophagectomy**				
*Days of in-hospitalisation (interquartile range)*	16 (1–39)	14 (2–37)	15 (4–44)	
*Number of hospitalisations (interquartile range)*	2 (1–4)	2 (1–4)	2 (1–4)	
*Number of outpatient visits (interquartile range)*	6 (3–13)	6 (2–16)	8 (4–18)	

^2 missing values.

*Only major complications causing clinical symptoms, as defined in the method section are included.

### Healthcare utilisation between discharge and one year after oesophagectomy

There was an increased total number of days of in-hospitalisation in patients with more than 1 complication from discharge to one year after oesophagectomy, compared to patients without complications (IRR 1.7, 95% CI 1.1–2.7) (Table 2). We found no association between number of complications and number of hospitalisations (IRR 1.3, 95% CI 0.9–1.8) or number of outpatient visits (IRR 1.2, 95% CI 1.0–1.4) (Table 2). The IRRs for patients with cardiovascular or cerebrovascular complications, compared to patients without complications, were 1.4 (95% CI 0.8–2.2) for total number of days of in-hospitalisation, 1.1 (95% CI 0.7–1.6) for number of hospitalisations, and 1.2 (95% CI 1.0–1.5) for number of outpatient visits (Table 2). The total number of days of in-hospitalisation was increased in patients with anastomotic insufficiency (IRR 2.0, 95% CI 1.1–3.4) (Table 2). The association with number of hospitalisations (IRR 1.4, 95% CI 0.9–2.2) and number of outpatient visits (IRR 1.3, 95% CI 1.0–1.6) for patients with anastomotic insufficiency was limited (Table 2).

**Table 2:** Healthcare utilisation in relation to major postoperative complications, cardiovascular or cerebrovascular complications and anastomotic insufficiency among 390 patients treated with curatively intended oesophagectomy for cancer who survived at least 1 year.

	< 1 year of oesophagectomy						≥ 1 year to 5 years of oesophagectomy					
	Inpatient				Outpatient		Inpatient				Outpatient	
	*Days of in-hospitalisation*		*Number of hospitalisations*		*Number of outpatient visits*		*Days of in-hospitalisation*		*Number of hospitalisations*		*Number of outpatient visits*	
	IRR	95%-CI	IRR	95%-CI	IRR	95%-CI	IRR	95%-CI	IRR	95%-CI	IRR	95%-CI
**Complications**												
*No complications*	1.0	(reference)	1.0	(reference)	1.0	(reference)	1.0	(reference)	1.0	(reference)	1.0	(reference)
*1 complication*	1.3	(0.9–2.1)	1.3	(0.9–1.7)	1.1	(0.9–1.3)	1.3	(0.8–2.0)	1.2	(0.8–1.8)	1.1	(0.9–1.5)
*>1 complication*	1.7	(1.1–2.7)	1.3	(0.9–1.8)	1.2	(1.0–1.4)	1.5	(1.0–2.4)	1.1	(0.7–1.6)	1.2	(0.9–1.6)
*Complications other than cardiovascular or cerebrovascular complications*	1.6	(1.1–2.4)	1.4	(1.0–1.9)	1.1	(0.9–1.3)	1.3	(0.9–2.0)	1.2	(0.8–1.7)	1.2	(0.9–1.5)
*Cardiovascular or cerebrovascular complication(s)*	1.4	(0.8–2.2)	1.1	(0.7–1.6)	1.2	(1.0–1.5)	1.5	(0.9–2.5)	1.2	(0.7–1.9)	1.1	(0.8–1.5)
*Complications other than anastomotic insufficiency*	1.4	(0.9–2.1)	1.2	(0.9–1.7)	1.1	(0.9–1.3)	1.3	(0.9–1.8)	1.2	(0.8–1.6)	1.1	(0.9–1.4)
*Anastomotic insufficiency*	2.0	(1.1–3.4)	1.4	(0.9–2.2)	1.3	(1.0–1.6)	1.8	(1.0–3.0)	1.2	(0.7–2.0)	1.3	(0.9–1.7)

Adjusted incidence rate ratios* (IRR) with 95% confidence intervals (CI) are presented.

*Results were adjusted for age, sex, tumour stage, comorbidity, histological type of tumour, hospital volume and neoadjuvant therapy.

### Healthcare utilisation 1–5 years after oesophagectomy

The total number of days of in-hospitalisation 1–5 years following oesophagectomy was higher in patients with more than 1 complication (IRR 1.5, 95% CI 1.0–2.4). We found no association between number of complications and number of hospitalisations (IRR 1.1, 95% CI 0.7–1.6) or number of outpatient visits (IRR 1.2, 95% CI 0.9–1.6) (Table 2). Compared to patients without complications, patients with cardiovascular or cerebrovascular complications had IRRs of 1.5 (95% CI 0.9–2.5) for total number of days of in-hospitalisation, 1.2 (95% CI 0.7–1.9) for number of hospitalisations, and 1.1 (95% CI 0.8–1.5) for number of outpatient visits. There was an increased total number of days of in-hospitalisation in patients with anastomotic insufficiency (IRR 1.8, 95% CI 1.0–3.0) (Table 2), but no associations with number of hospitalisations (IRR 1.2, 95% CI 0.7–2.0) or number of outpatient visits (IRR 1.3, 95% CI 0.9–1.7) after these complications (Table 2).

## Discussion and Conclusion

This study indicates a long-lasting increase of healthcare utilisation, especially for total duration of in-hospitalisation, among patients who suffer from a major complication shortly after oesophagectomy for cancer, particularly among patients with anastomotic insufficiency.

Strengths of this study include the prospective and population-based design with high participation rate with long and complete follow-up of the patients. Moreover, the information on complications was comprehensive and based on clear definitions formulated before the initiation of the data collection, and the assessment of both the inpatient and outpatient healthcare utilisation was objective and accurate, ensuring high validity of both exposure and outcome measures. Our used outcome measures, number of hospitalisations, hospitalisation days and number of outpatient visits, are frequently used proxies of healthcare utilisation costs since these also enable comparison between countries with other healthcare and reimbursement systems. Also, the results were adjusted for all major prognostic factors, although residual confounding cannot be excluded in observational research. By using quasi-Poisson models, we took overdispersion into account to assess for potential clustering. Although we could have used several other statistical models for the analyses, more complex models such as generalised estimation equation models (GEEs) showed very similar results.

Among weaknesses was the limited statistical power to detect weak differences between groups. Yet, the study included virtually all eligible patients in Sweden (limiting the risk of selection bias), and moderately strong associations were found. At the time this study was conducted (2001–2005), there was insufficient evidence for the efficacy of neoadjuvant or adjuvant oncological therapy in patients operated for oesophageal cancer with curative intent, and thus this therapy was not used in Sweden during the study period. Only a small proportion of patients received neoadjuvant treatment, which was not current practice for patients operated for oesophageal cancer at that time in Sweden. Although we corrected for potential confounding by neoadjuvant therapy, our findings may differ from cohorts receiving other neoadjuvant or adjuvant treatment regimens. Other scoring systems for complications were not available at the time for initiating the present cohort, for example including different levels of severity.[20]. However, we chose to only include major complications considered relevant for this type of surgery, as defined before the data-collection by a board of Swedish oesophageal surgeons.[14,16] Post-hoc comparison with the Accordion Severity Grading System shows that our definitions of complications would most likely fall within the categories 3–5 (“Severe”), although it was not always possible to align these definitions.[21] The classification of comorbidities was decided upon in a similar manner and does not enable post-hoc re-categorisation to other comorbidity scores such as the Charlson comorbidity index,[22] using slightly other categories, definitions and additional comorbidities which were not collected as such in the present study. However, since comorbidities are only examined as confounders and not as exposures, the impact of the used categorisation was considered limited. Another potential weakness was that we did not restrict our analyses to cancer-specific healthcare utilisation. Distinguishing cancer-specific and other healthcare utilisation is not always straight forward. Overall health may remain worse compared to the general population as a consequence of the cancer or treatment, consequently indirectly increasing primary and specialised health care utilisation.[23,24] There was little difference in presence of comorbidities between patients without complication and patients with one complication, but approximately 10% more comorbidities were found in the group with more than one complication. However, the amount of non-cancer related specialised medical healthcare utilisation, in particular hospitalisations, is assumed to be rather similar in the comparison groups of patients. Thus, any misclassification of the outcome was likely to be non-differential, and would therefore rather dilute the associations between the complications and healthcare utilisation, which would indicate that the true associations might be stronger.

To the best of our knowledge, this is the first study that has explored the relation between postoperative complications and long-term healthcare utilisation after oesophagectomy. Overall, we found an increase in healthcare utilisation in relation to postoperative complications in the long-term after oesophagectomy, at least regarding the total number of days of in-hospitalisation. Healthcare utilisation was particularly associated with an increase in patients with anastomotic insufficiency. However, other factors, such as tumour stage and treatment-related factors may also influence the risk of complications and long-term healthcare utilisation. Since these outcomes have been investigated only for this cohort, more studies are necessary to confirm the findings.

In conclusion, this population-based and prospective cohort study indicates that early major complications following oesophagectomy for cancer may be associated with long-term healthcare utilisation, as indicated by an increased total in-hospitalisation period, especially in patients who have had anastomotic insufficiency.
